# Bio-Inspired Proprioception for Sensorless Control of a Klann Linkage Robot Using Attention-LSTM

**DOI:** 10.3390/biomimetics11030192

**Published:** 2026-03-05

**Authors:** Hoejin Jung, Woojin Choi, Sangyoon Woo, Wonchil Choi, Won-gyu Bae

**Affiliations:** Department of Electrical Engineering, Soongsil University, Seoul 06978, Republic of Korea; wjdghlwls1234@soongsil.ac.kr (H.J.); dnwlsrnfl@soongsil.ac.kr (W.C.); wsy04018@soongsil.ac.kr (S.W.); dnjsclf1456@soongsil.ac.kr (W.C.)

**Keywords:** biological proprioception, artificial intelligence, angle prediction, low-cost, legged robot

## Abstract

While walking robots possess significantpotential for various real-world applications, the reliance on high-performance sensors and complex control architectures for precise gait control remains a significant barrier to commercialization and lightweight design. To overcome these engineering limitations and lay the groundwork for a sensing paradigm adaptable to complex terrains, this study proposes an AI-based sensorless feedback control framework that incorporates the biological principles of proprioception. To this end, a walking robot leveraging the morphological intelligence of the Klann linkage was developed. We constructed a time-series dataset by defining motor current signals as ‘interoceptive sensing’ information—analogous to biological muscle feedback—and synchronizing them with absolute angular data. This dataset was used to train an Attention-LSTM (A-LSTM) model, which predicts future motor states in real-time by decoding nonlinear physical information embedded within internal current data, independent of external environmental sensors. By integrating the proposed model into a PI controller, a stable biomimetic walking loop was successfully implemented without the need for additional position sensors.

## 1. Introduction

Walking robots exhibit exceptional mobility efficiency in real-world environments characterized by rough terrain and complex obstacles, offering significant potential across diverse fields such as disaster response, defense surveillance, and agricultural automation. Recently, sophisticated model-based paradigms have been proposed in the field of hydraulic legged robotics to precisely control the complex nonlinear dynamics of these systems. For instance, Fang et al. innovatively enhanced joint force estimation precision through a Fast Direct Adaptive Robust Control (FDARC) strategy based on real-time parameter estimation [1]. The WLR-3P robot achieved high-speed locomotion and environmental adaptability simultaneously using a hierarchical distributed control system [2]. Furthermore, Zhang et al. demonstrated effective management of closed-chain dynamics by introducing a Virtual Decomposition Control (VDC) framework [3].

Despite their outstanding dynamic performance, these state-of-the-art (SOTA) strategies share a common limitation: they rely entirely on high-performance sensor arrays—such as encoders, pressure sensors, and Inertial Measurement Units (IMUs)—and require substantial computational resources for recursive model calculations [4]. These requirements not only increase system costs and manufacturing complexity, but also act as a critical bottleneck in achieving the system lightweighting and simplification essential for biomimetic robots. Moreover, the proliferation of sensors entails complicated wiring and calibration processes, resulting in structural limitations that degrade the robot’s environmental adaptability and maintenance efficiency [5].

Biological organisms in nature offer profound inspiration for overcoming these engineering limitations. In contrast to the aforementioned high-cost, sensor-centric paradigms, animals regulate their gait and perceive their posture in real-time through proprioception—processing muscle contraction and tension signals—without having to visually monitor every leg position [6]. In other words, they adapt to complex terrains solely through the intelligent interpretation of internal signals, devoid of high-precision external sensors. Consequently, there is a growing need in the engineering domain for low-cost, high-efficiency sensor-less control structures that substitute high-precision external sensors or complex numerical models with electrical signals from within the motors, treated as sensory information [7].

Inspired by these biological mechanisms, this study proposes an artificial intelligence-based sensor-less feedback control framework that estimates actual motor angles and utilizes them for control using only low-cost current sensors. The current signals generated by the motor serve as internal feedback reflecting driving torque and mechanical load conditions, functionally resembling the mechanism where muscle tension and load information are processed as proprioceptive signals in biological systems [8,9]. However, since current signals are indirect, noisy, and nonlinear time-series data, we integrated a sophisticated time-series analysis model based on Attention-LSTM (A-LSTM) to accurately map these signals to physical states [10,11].

This approach fundamentally diverges from traditional sensorless control methods that primarily focus on the physical modeling of current–torque correlations, as well as high-performance legged robot systems relying on complex analytical frameworks such as Virtual Decomposition Control (VDC) or Adaptive Robust Control (ARC) [12]. Rather than treating motor current as a mere variable for torque estimation, this study redefines it as a ‘cognitive feedback signal’ for real-time postural awareness, aiming to implement data-driven, bio-inspired self-perception.

By utilizing the feature extraction capabilities of the A-LSTM model instead of model-based strategies that demand heavy computational loads and precision sensing, we establish a robust ‘proprioceptive loop’ that operates without expensive encoders. This ensures both the lightweight design and the practical utility of the system. To this end, this study integrated a Klann linkage-based walking mechanism with an A-LSTM prediction model to validate its effectiveness. The Klann linkage provides morphological intelligence by enabling complex walking patterns with only a single driving axis, structurally addressing the control complexity issues typically encountered by traditional multi-degree-of-freedom robots [13].

As a foundational step for adaptation to real-world environments, this study experimentally verified the system’s recognition and recovery capabilities—even on flat terrain—through the injection of forced physical disturbances such as “stuck states”. Notably, the autonomous recovery demonstrated under unexpected physical constraints is the result of engineering the reflex-like mechanisms found in living organisms [14]. In summary, this study demonstrates the potential of intelligent sensorless control to replace expensive state-of-the-art (SOTA) control strategies, serving as a critical engineering cornerstone for the future design of economic and robust robotic systems capable of adapting to unstructured environments.

## 2. System Overview and Key Components

### 2.1. Klann Linkage

The Klann linkage is a planar mechanism widely utilized in the implementation of legged robots. It is a six-bar linkage structure designed to generate a walking trajectory resembling animal locomotion through a single rotary input. Unlike traditional crank-slider mechanisms that produce linear leg motion, the Klann linkage naturally generates distinct support and swing phases suitable for locomotion, determined by the length ratios of the links and the geometric arrangement of the pivot points [15]. These kinematic characteristics allow for the Klann linkage to replicate ground contact patterns similar to biological gaits, thereby enhancing the robot’s terrain adaptability and locomotive stability.

Furthermore, because the Klann linkage can generate a complete leg trajectory using a single actuator, it offers a significantly simpler structure and lower manufacturing costs compared to conventional legged robots that require multi-degree-of-freedom (DOF) joints [16]. This presents a critical advantage by minimizing complex actuator configurations, expensive sensors, and precise alignment processes—factors that typically act as major constraints to the commercialization of legged robots. Specifically, in the context of establishing the low-cost feedback control architecture proposed in this study, the linkage-based mechanism reduces the dimensionality of control variables, thereby significantly lowering both the complexity of controller design and the computational burden. Recent studies have similarly demonstrated that linkage-based transmission systems can effectively replicate biological kinematics while reducing control dimensionality [17].

Leveraging these structural advantages, we constructed a walking robot based on the Klann linkage and monitored the locomotion state utilizing current data from the motor attached to the linkage’s pivot axis. The regular mechanical motion of the linkage induces periodic and distinct patterns in the motor current signals. These patterns serve as a crucial basis for the AI model to learn temporal dynamics and predict future motor angles. In other words, the repetitive yet non-linear gait patterns of the Klann linkage align naturally with the time-series analysis (A-LSTM) model employed in this study, facilitating stable locomotion control without the need for additional angular sensors.

**Figure 1:**
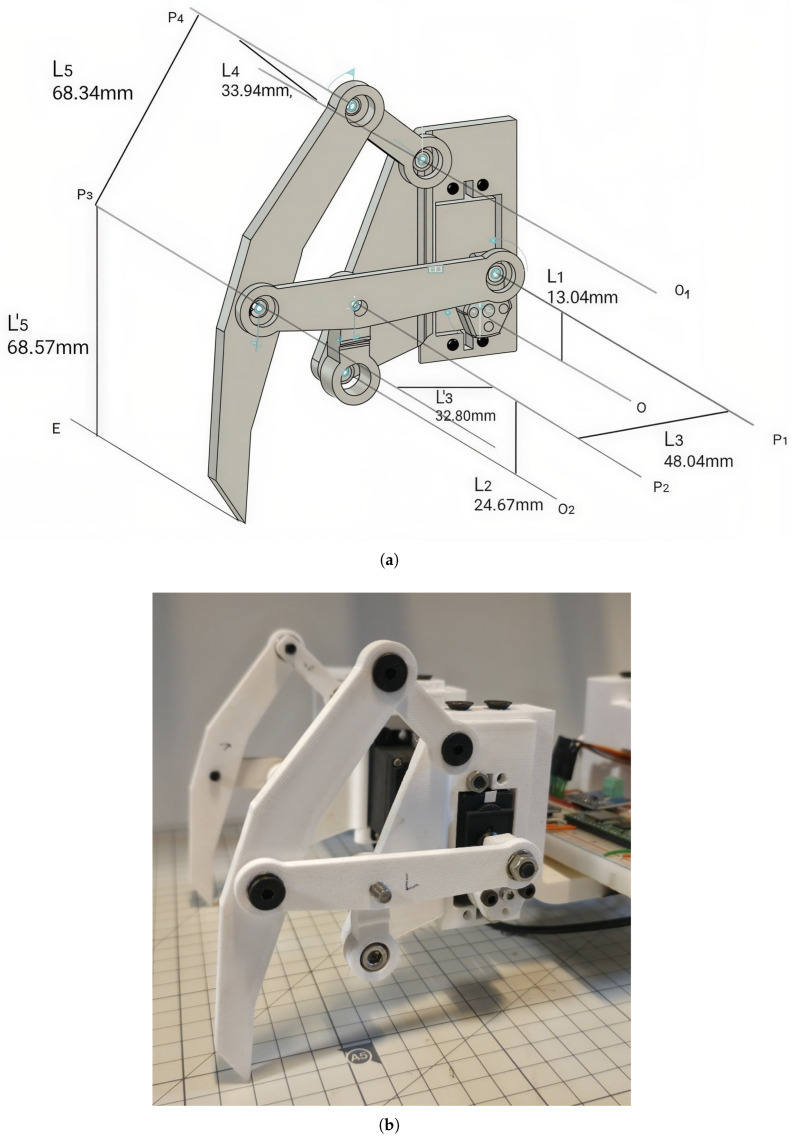
(**a**) Modeled legged with 3D CAD Program(Fusion-360) (**b**). Implementation with 3D printers for the corresponding modeling.

Thus, the Klann linkage serves as more than a mere mechanical apparatus; it is a core component that aligns perfectly with the objective of this study: to ensure practical locomotion performance while maintaining a low-cost, low-complexity architecture. To implement the mechanism, the linkage was designed using Autodesk Fusion 360 and fabricated via 3D printing, as illustrated in Figure 1.

#### 2.1.1. Kinematic Modeling

**Figure 2:**
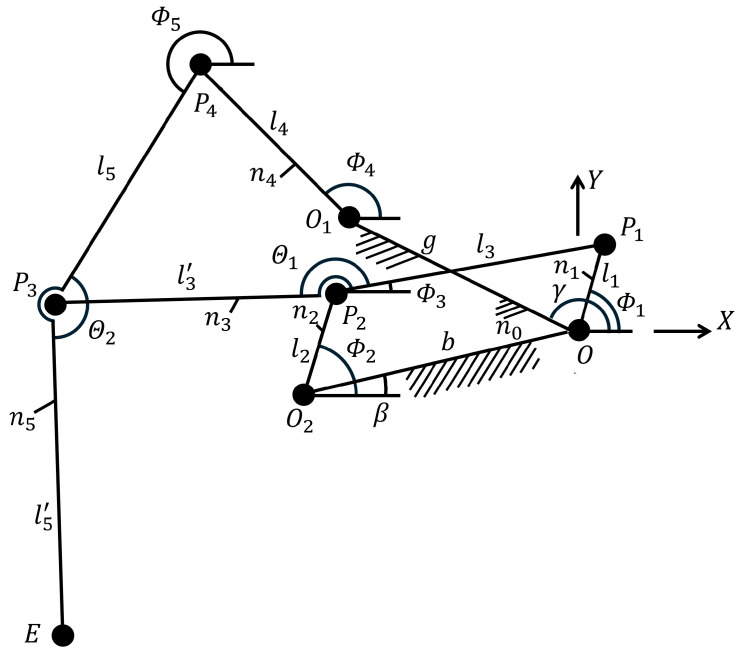
Klann linkage for mathematical modeling.

To optimize the robot’s locomotion, it is essential to establish the kinematic relationship between the driving crank and the leg endpoints. The linkage assembly comprises a crank (designated as the actuator), a connecting rod, and rockers interconnected via pivot joints. Figure 2 illustrates the simplified linkage structure utilized for mathematical modeling.

The coordinates of the endpoint E(x,y), which determine the gait trajectory, are derived from the geometric constraints of the links and the input angle. Based on the vector loop equations, the position of the endpoint *E* can be expressed as follows:

(1) $$E_{x}=-b\cdot \cos\beta +l_{2}\cdot \cos\phi_{2}+l_{3}^{\prime }\cdot \cos\left(\theta_{1}+\phi_{3}\right)+l_{5}^{\prime }\cdot \cos\left(\phi_{5}+\pi -\theta_{2}\right)$$

(2) $$E_{y}=-b\cdot \sin\beta +l_{2}\cdot \sin\phi_{2}+l_{3}^{\prime }\cdot \sin\left(\theta_{1}+\phi_{3}\right)+l_{5}^{\prime }\cdot \sin\left(\phi_{5}+\pi -\theta_{2}\right)$$

Here $l_{n}$ represents the length of each link, and the angle is determined by the instantaneous direction of the pivot relative to the reference frame.

#### 2.1.2. Trajectory Optimization and Parametric Specification

In a preliminary study related to this work, the link length ratios were optimized using a Genetic Algorithm (GA) to ensure stable and efficient locomotion. The optimization prioritized the flattening of the ground-contact phase to minimize vertical oscillations. The objective function was defined as the Sum of Squared Errors (SSEs) between the target flat-ground height ($h_{\mathrm{target}}$) and the simulated endpoint height ($h_{i}$) during the contact interval [18].

(3) $$SSE=\sum_{i=70}^{110}{\left(h_{i}-h_{target}\right)}^{2}$$

Following an evolutionary search spanning 10,000 generations, the optimal link parameters were determined to achieve the highest locomotive stability. The specific dimensions and fixed-angle configurations of the fabricated links are summarized in Table 1.

### 2.2. Current Sensor & A-LSTM

For the control of the walking robot, we adopted an approach that monitors motor states utilizing low-cost current sensors, thereby eschewing expensive position and attitude sensors such as encoders or Inertial Measurement Units (IMUs). Current sensors offer a high information-to-cost ratio because their electrical signals fluctuate directly in response to variations in motor load, the mechanical kinematics of the linkage, and ground contact events. In particular, the current waveforms generated during locomotion exhibit both periodicity and non-linear characteristics, offering the distinct advantage of allowing for the indirect estimation of motor states, such as rotational angle and load conditions.

The current data constitutes time-series data measured continuously from the initiation of actuation, with patterns that dynamically evolve according to the robot’s gait phase and linkage position. Given that these dynamic characteristics preclude accurate state estimation using simple static models, a deep learning-based model capable of learning the temporal correlations within the time-series data is required.

To address this, this study utilizes an Attention-based Long Short-Term Memory (A-LSTM) model. While standard LSTM networks resolve the long-term dependency problem inherent in traditional Recurrent Neural Networks (RNNs) and effectively learn long-term patterns in time-series data, they assign uniform weights to all time steps [19]. Consequently, standard LSTMs often yield suboptimal performance when processing signals like motor current, which are characterized by irregular fluctuations or sudden spikes in information density at specific intervals.

The A-LSTM overcomes this limitation by integrating an attention mechanism, which assigns higher weights to time steps that are significantly relevant for prediction within the time-series input [20,21]. This enables the model to more accurately capture local transients in the current data—such as ground contact, load surges, and linkage transition points—thereby facilitating more precise estimation of future motor angles. In particular, since the Klann linkage mechanism inherently induces repetitive load variations at specific kinematic phases, it aligns exceptionally well with the selective feature extraction capabilities of the A-LSTM.

In this study, the A-LSTM was trained using time-series data collected from current sensors at fixed intervals (0.1 s) as input, with actual motor angles obtained via camera-based labeling serving as the ground truth targets. The trained model generates predicted angles in real-time and is integrated with the PI controller implemented in this study, establishing a sensorless feedback control architecture that maintains gait stability without additional sensors.

#### LSTM Cell Dynamics and Attention Mechanism

Given an input current sequence ${\left\{x_{t}\right\}}_{t=1}^{T}$, the LSTM cell learns long-term dependencies through its gated architecture and sequentially updates the hidden state. The operations at each time step *t* are as follows:

(4) $$\begin{array}{cc} i_{t} & =\sigma (W_{i}\left[h_{t-1},x_{t}\right]+b_{i}),\\ f_{t} & =\sigma (W_{f}\left[h_{t-1},x_{t}\right]+b_{f}),\\ o_{t} & =\sigma (W_{o}\left[h_{t-1},x_{t}\right]+b_{o}),\\ {\tilde{C}}_{t} & =\tanh(W_{C}\left[h_{t-1},x_{t}\right]+b_{C}),\\ C_{t} & =f_{t}\cdot C_{t-1}+i_{t}\cdot {\tilde{C}}_{t},\\ h_{t} & =o_{t}\cdot \tanh\left(C_{t}\right) \end{array}$$

where $i_{t},f_{t},o_{t}\in R^{d_{h}}$ denote the input, forget, and output gate vectors, respectively; $C_{t}\in R^{d_{h}}$ represents the cell state; and $h_{t}\in R^{d_{h}}$ is the hidden state. The LSTM generates a set of hidden states $\left\{h_{1},h_{2},\ldots ,h_{T}\right\}$ for the entire sequence.

To learn the relative contribution of each hidden state to the final prediction, an attention mechanism is integrated with the LSTM. First, the attention score for each hidden state is calculated using the following equation:

(5) $$e_{i}=u\cdot \tanh\left(W\cdot h_{i}+b\right)$$

where $e_{i}$ is a scalar value representing the importance of the *i*-th time step, and $W\in R^{d_{a}\times d_{h}}$ and $u\in R^{d_{a}}$ are learnable parameters. Next, the attention weights are computed using the Softmax function to ensure they form a normalized probability distribution:

(6) $$\alpha_{i}=\frac{\exp(e_{i})}{\sum_{j=1}^{T}\exp\left(e_{j}\right)},\sum_{i=1}^{T}\alpha_{i}=1$$

The final context vector *c* is obtained by the linear combination of these weights and the hidden states:

(7) $$c=\sum_{i=1}^{T}\alpha_{i}h_{i}$$

**Figure 3:**
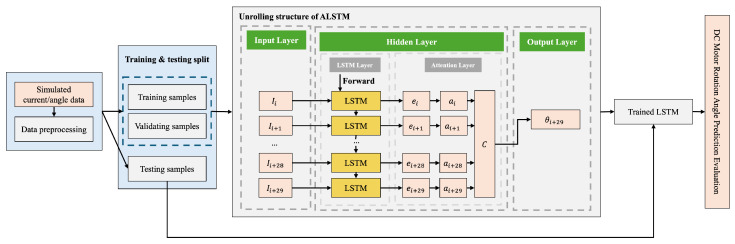
Architecture of the A-LSTM model.

The context vector *c*, derived through this process, is fed into a fully connected layer to predict the final output value:

(8) $$\hat{\theta }=W_{o}^{\left(out\right)}c+b_{o}^{\left(out\right)}$$

where $\hat{\theta }$ represents the predicted actual motor angle, and $W_{o}^{\left(out\right)}$ and $b_{o}^{\left(out\right)}$ are the learnable parameters of the output layer. The detailed architecture of the A-LSTM model utilized in this study is illustrated in Figure 3 [18].

## 3. Methodology

### 3.1. Collect Current Data

To train the proposed A-LSTM-based predictive model, a time-series dataset that captures the correlation between motor current variations and actual angular positions is essential. To this end, motor current data were acquired while operating the Klann linkage-based walking robot under open-loop conditions with constant actuation cycles. The rationale for employing the open-loop driving method was to record intrinsic current patterns resulting from pure mechanical load variations and locomotion dynamics, devoid of any compensatory influence from a controller. This approach ensures that characteristic fluctuations in the current signal—such as periodic load variations, ground contact timing, and transitions between swing and stance phases—are accurately reflected in the training dataset.

Current data were sampled at 0.1 s intervals during motor operation, with each data point annotated with an absolute timestamp to record the precise acquisition time. This timestamping is a critical prerequisite for accurate alignment with camera-based angular labels, ensuring precise time synchronization between the sensor data and video imagery.

To obtain the actual absolute angles (target angles), a video-based data acquisition process was conducted in parallel, filming the motor within the same experimental environment. The motor horn of the robot was continuously recorded during locomotion to store frame-by-frame video data. Each video frame was assigned temporal metadata to facilitate alignment with the current data along the same time axis. In the initial setup phase, the position of the motor horn and a reference line were manually labeled using Label Studio. These labels served as the basis for an automated angle extraction algorithm, which subsequently calculated the absolute motor angles directly from the video footage.

Through this process, each video frame was converted into an angular value with absolute timing information. These values were then matched with the current data timestamps to generate the final time-series dataset of current-angle pairs. This dataset serves as the foundation for training the A-LSTM model, enabling it to effectively learn the non-linear relationships between current variations and motor states.

### 3.2. Training the A-LSTM Model with Current and Angle Data

Since the time-series current data collected from sensors and the absolute motor angle data acquired via the camera system are obtained through independent channels, a precise time alignment process is a prerequisite for utilizing them in training. In this study, the two signal sources were aligned based on absolute timestamps common to both datasets. Each recorded current sample was paired with the absolute angle of the temporally nearest video frame, thereby generating a final time-series table that reflects the current-to-angle relationship. Any temporal discrepancies arising during this matching process were minimized using linear interpolation to guarantee the continuity of the model input.

The resulting dataset comprises current time-series windows as input features and the corresponding absolute motor angles at specific time steps as target values, which are directly utilized for training the A-LSTM model. The length of the input window was configured to sufficiently encompass the periodicity and load variation patterns inherent in the current waveforms, with the model structured to predict future motor angles based on historical current variations.

The A-LSTM model incorporates an attention mechanism atop standard LSTM layers, assigning greater weight to temporal segments within the input window that are critical for prediction. The attention layer automatically learns significant inflection points in the current signal—such as ground contact events, linkage rotation transitions, and sudden load changes—thereby achieving superior angle prediction performance compared to conventional LSTMs. Training was conducted to minimize the discrepancy between actual and predicted angle values, utilizing Mean Squared Error (MSE) as the primary loss function.

To evaluate generalization performance, the entire dataset was partitioned into training and validation sets. Furthermore, regularization techniques, including dropout and early stopping, were applied during the training process to prevent overfitting. Ultimately, the trained A-LSTM model is capable of generating future motor angles in real-time using solely current data inputs. These predicted values are subsequently transmitted to the PI controller, establishing a low-cost, sensorless feedback control loop.

#### Dataset Visualization and Correlation Analysis

**Figure 4:**
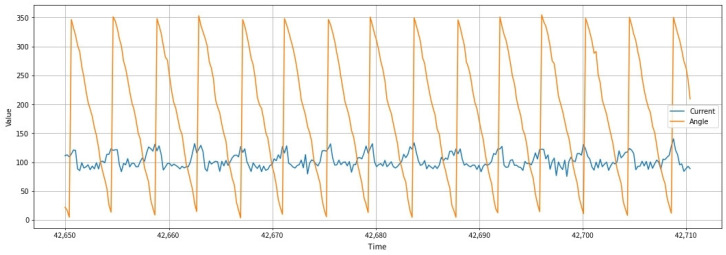
Temporal correlation between motor current signatures and crank angle variations under mechanical load of the Klann linkage.

To substantiate the authenticity and efficacy of the proposed sensorless feedback control framework, we applied the data-driven modeling process established in the prior study to our robotic system and visualized the key performance indicators. Figure 4 illustrates the characteristics of the raw dataset collected during the robot’s locomotion. The motor current signatures exhibit distinct periodic patterns that fluctuate in synchronization with the mechanical load variations of the Klann linkage. Notably, these current fluctuations show a strong temporal correlation with the linear changes in the crank angle, providing a robust empirical basis for the A-LSTM model to map electrical signals to physical states.

**Figure 5:**
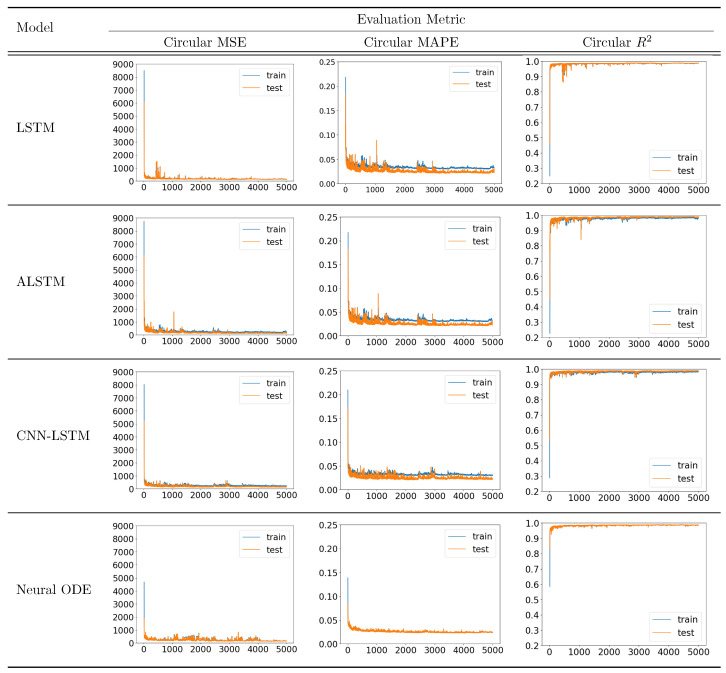
Convergence curves of evaluation metrics for assessing the learning stability and predictive performance of different neural network models.

The training convergence was monitored to evaluate the learning stability of the proposed architecture. As demonstrated in Figure 5, the A-LSTM model, utilizing the customized Circular MSE loss function, exhibited superior convergence behavior starting from the early stages of training. Compared to other deep learning candidates such as standard LSTM and CNN-LSTM, the A-LSTM achieved the lowest validation error, effectively minimizing the discrepancy between predicted and actual values through its attention mechanism.

**Figure 6:**
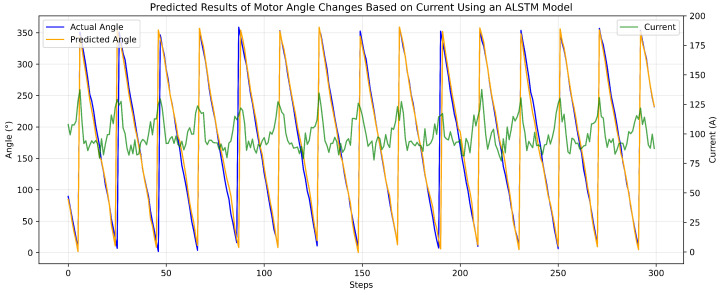
Predicted results of motor angle changes based on current an ALSTM model.

To verify the quality of the fit and the overall accuracy of the state estimation, the predicted motor angles were compared directly against the ground truth labels obtained via camera-based measurements. Figure 6 shows the alignment between the predicted results and the actual angle changes. The prediction waveform accurately follows the nonlinear dynamics and phase transitions of the motor rotation with minimal deviation. This high-fidelity fit visually demonstrates that the intelligent interpretation of internal current signatures can reliably replace high-cost external encoders for real-time robotic proprioception.

### 3.3. PI Control and ALSTM Tuning

A linear Proportional-Integral (PI) controller was implemented to maintain stable locomotion based on the motor angles predicted by the A-LSTM model. The PI control strategy is characterized by its structural simplicity and low computational overhead, rendering it highly suitable for small-scale robotic platforms that require real-time control. Furthermore, it offers the distinct advantage of providing stable response characteristics for the single control variable associated with the motor’s rotational axis.

#### 3.3.1. PI Controller Parameters

The objective of the PI controller is to perform phase synchronization between the reference motor ($Ch_{0}$) and the follower motors ($Ch_{1}$–$Ch_{3}$) for the cooperative control of the multi-jointed actuation system. To this end, a discrete-time controller operating at a frequency of 10 Hz ($T_{s}=0.1$ s) was designed within the microcontroller.

The goal of the controller is to modulate the Pulse Width Modulation (PWM) duty cycle such that the current angle $\theta_{meas}$ of the follower motors tracks the target angle $\theta_{target}$ generated by the reference motor with zero steady-state error. The target phase relationship for each motor is defined as shown in Equation (9).

(9) $$\theta_{target,i}=\left\{\begin{array}{cc} \theta_{ref} & \mathrm{for}\;i\in \{0,3\}\\ \left(\theta_{ref}+180^{{}^{\circ}}\right)\;\left(\mathrm{mod}\;360^{{}^{\circ}}\right) & \mathrm{for}\;i\in \{1,2\} \end{array}\right.$$

Here, $\theta_{ref}$ denotes the reference phase received from an external client. To address the discontinuity occurring at the $0^{{}^{\circ}}$/$360^{{}^{\circ}}$ boundary inherent to rotating bodies, the angular error e[k] is calculated within the range of $\left[-180^{{}^{\circ}},180^{{}^{\circ}}\right]$ as shown in Equation (10).

(10) $$e\left[k\right]=(\left(\theta_{meas}-\theta_{target}+180\right)\;\mathrm{mod}\;360)-180$$

To prevent abrupt control inputs caused by sensor noise and communication latency, the calculated error is processed through a first-order Low-Pass Filter (LPF) as expressed in Equation (11). In this study, the filter coefficient α was set to 0.3 to mitigate rapid fluctuations in the error signal.

(11) $$e_{filt}\left[k\right]=\alpha \cdot e\left[k\right]+\left(1-\alpha \right)\cdot e_{filt}\left[k-1\right]$$

Finally, the PWM duty cycle adjustment u[k] for motor speed control is determined according to the PI control law defined in Equation (12).

(12) $$u\left[k\right]=-C_{scale}\left(K_{p}\cdot e_{filt}\left[k\right]+K_{i}\sum_{n=0}^{k}e_{filt}\left[n\right]\cdot T_{s}\right)$$

Here, $C_{scale}$ is a scaling constant used to convert the angular error into a duty cycle percentage (%), which was set to 0.10 (%/deg). The key parameters employed to ensure the stability of the control system are listed in Table 2.

Since $Ch_{0}$ serves as the provider of the reference phase, its control gains were set to zero, allowing it to operate in an open-loop manner or strictly follow upper-level commands. Conversely, $Ch_{1}$ through $Ch_{3}$ actively compensate for deviations from the reference phase using the configured $K_{p}$ and $K_{i}$ values. Furthermore, to prevent hardware damage resulting from excessive control inputs, the duty cycle change per iteration was restricted to ±1.0% (Slew Rate Limiting), and safety mechanisms were implemented to ensure operation strictly within the physically permissible range (Clamping) for each channel.

#### 3.3.2. ALSTM Hyperparameter Tuning and Training Configuration

For model optimization and tuning, this study employed a composite loss function (Combined MSE Loss) that reflects the periodic characteristics of the angular data. Training was conducted by combining standard Mean Squared Error (MSE) with Circular MSE, which accounts for the cyclic nature of the angles, as defined in Equation (13).

(13) $$L_{total}=\left(1-\alpha \right)\cdot L_{MSE}+\alpha \cdot L_{CircularMSE}$$

Here, α was set to 0.5 to assign equal weight to both error terms. To enhance training stability and convergence speed, a linear decay learning rate scheduling strategy was applied; the learning rate was linearly decreased from an initial value of $5\times 10^{-5}$ to $1\times 10^{-5}$ over 5000 epochs. Detailed hyperparameter configurations are presented in Table 3.

### 3.4. Modeling Framework and Assumptions

For the sake of methodological transparency and to ensure the reproducibility of the proposed sensorless control framework, the key parameters and physical assumptions employed in this study are summarized in Table 4. This comprehensive overview encompasses the mechanical specifications of the Klann linkage, the electrical characteristics of the sensing system, and the hyperparameter configurations for the A-LSTM model.

By centralizing these parameters, we clarify the boundary conditions under which the A-LSTM model operates. The inclusion of physical assumptions, such as the rigid body behavior of the 3D-printed components and the stability of the supply voltage, further strengthens the theoretical rigor of the proposed data-driven proprioceptive loop.

## 4. Experiments and Results

This section details the experimental procedures conducted to validate the effectiveness of the proposed A-LSTM-based sensorless feedback control system and presents the corresponding results. As a foundational step for adaptation to unstructured real-world environments, this experiment focuses on assessing how precisely the system can perceive and overcome physical constraints using only internal ‘interoceptive sensing’ signals, without reliance on external sensors. The experiments were conducted by establishing control and comparative models based on the application and type of control algorithms. Three distinct models were defined: (1) the “Zero Model,” representing an open-loop state without any feedback control; operating in an open-loop state without feedback control; (2) the “Rule-based Model,” which induces reactive responses at specific points based on predefined current thresholds; and (3) the “Main Model,” the framework proposed in this study, which utilizes the A-LSTM to interpret nonlinear physical information embedded in current data to predict future motor states, which are then integrated with a PI controller.

The comparative Rule-based Model was designed based on the correlation between motor current and output torque. Specifically, the moment when the motor current reaches its peak was defined as the instant the robot applies maximum propulsion force to the ground. Accordingly, intervals where the real-time current values were measured within the 75% range (0.75 quantile) of the experimental data were identified as valid ground support and propulsion phases. The system was configured to activate the control logic by returning the angle data from these specific intervals for a predetermined duration. The Main model was operated by loading the weight files trained on the previously established 120,000-point dataset into the control system. The data were recorded at a sampling frequency of 10 Hz, corresponding to a total recording duration of 200 min. Given the robot leg’s average gait cycle of 3.2 s, the dataset encompasses approximately 3750 gait cycles, capturing a diverse range of physical interactions and load variations. To ensure robust model development and objective performance evaluation, the dataset was partitioned into training, validation, and testing sets at an 8:1:1 ratio (96,000, 12,000, and 12,000 points, respectively). The training and validation sets were utilized for weight optimization and hyperparameter tuning, whereas the testing set was reserved exclusively for evaluating the model’s final predictive accuracy and generalization capability. All three models executed locomotion tasks under identical physical environmental conditions with controlled external variables.

Evaluation metrics were configured to provide a multifaceted analysis of the robot’s gait stability, mobility, and long-term operational reliability. First, an intentional physical disturbance termed “stuck state”—a condition where forward motion is impeded due to abnormal leg synchronization—was artificially induced. This simulates a scenario where the robot’s legs are mechanically restricted by physical boundaries, representing the most severe physical constraint the system might encounter even on flat terrain. We then measured the escape time required for the control algorithm to autonomously detect this state and restore normal locomotion. Additionally, the traversal time for a 1 m straight section was measured to evaluate basic locomotion efficiency. To assess the ability to maintain a stable trajectory over extended periods, a 2 min continuous walking test was conducted, analyzing the final distance traveled and path deviation. Finally, by analyzing lateral deviation components during locomotion, we comprehensively verified how effectively the proposed controller compensates for uneven ground friction or mechanical discrepancies to maintain directional stability and rectilinearity.

**Figure 7:**
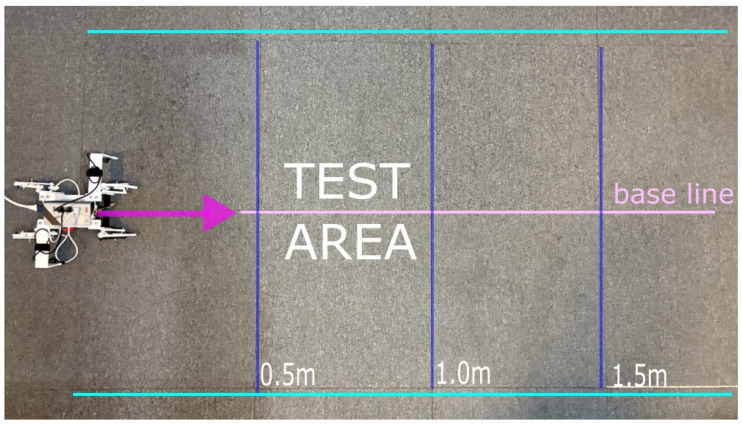
Experiment environment built using tiles.

To facilitate real-time monitoring of the movement trajectory and distance, a test grid was constructed using 0.5×0.5 m tiles, as shown in Figure 7, upon which the walking tests were performed. This baseline validation aims to demonstrate the effectiveness of the proposed recognition mechanism, proving that a high-level perception-control loop can be formed solely through the intelligent interpretation of current signals, without the need for expensive external sensors.

### 4.1. Control Verification and Walking Efficiency Analysis

The primary objective of this experiment is to ascertain whether the robot can autonomously maintain its posture and stably generate consistent gait patterns during locomotion utilizing solely estimations derived from low-cost current sensors, thereby eliminating the need for expensive position sensors such as encoders or IMUs.

Evaluation metrics were defined as the capability to autonomously escape from unexpected “stuck states” during locomotion and the ability to maintain directional trajectory stability despite external disturbances such as ground friction. The results of ten 1 m straight-line walking trials, as summarized in Table 5, indicate that the uncontrolled “Zero Model” recorded “Failed to Reach” outcomes in the majority of trials (Trials 2, 3, 5, 6, 7, 8, and 10), failing to arrive at the target destination.

Although the Rule-based Model demonstrated improved locomotion performance relative to the Zero Model, successfully reaching the destination in 8 trials, it exhibited limitations by failing to overcome the stuck state in specific instances (Trials 3 and 7). In contrast, the proposed Main Model successfully reached the destination in all ten trials, demonstrating superior robustness.

**Figure 8:**
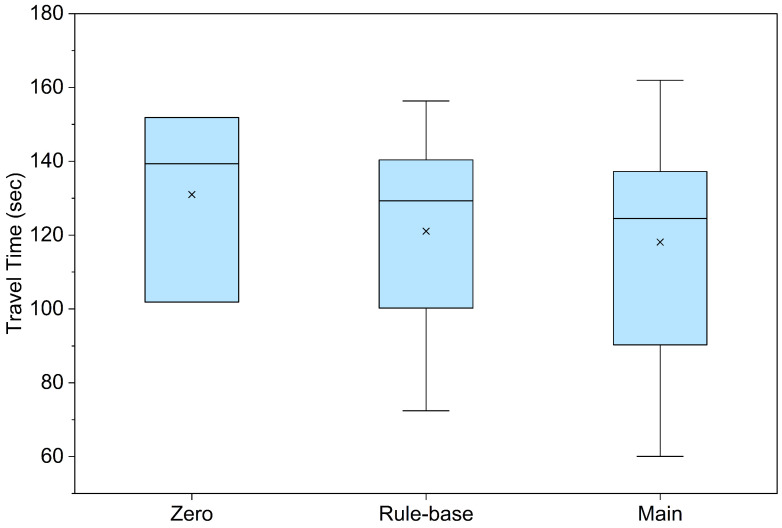
Box plot graph of Travel Time data. Symbol x represents the Mean. Detailed numerical mean and standard deviation (SD) statistics are provided in Table 5.

The performance superiority of the proposed model is further corroborated by the travel time distribution presented in Figure 8. The Main Model recorded the lowest mean and median values compared to the control groups. It achieved an overall average time of 118.11 s, representing a reduction of approximately 5.5% compared to the Rule-based Model (which averaged 121.08 s in successful trials). Furthermore, a paired *t*-test was conducted to verify the statistical significance considering the number of trials, revealing that the performance improvement of the Main Model is statistically significant (p=0.0062<0.01). These results imply that the proposed method ensures both rapid and stable mobility.

These findings suggest that the proposed control algorithm effectively ensures gait stability and enhances propulsion transfer by adaptively compensating motor output in response to real-time variations in ground friction and mechanical loads.

### 4.2. Evaluation of Stuck State Escape and Recovery Performance

Multi-legged walking robots are susceptible to a synchronization phenomenon wherein the motions of motors driving individual legs become abnormally aligned due to unexpected external disturbances or ground friction during locomotion. When the legs operate simultaneously rather than sequentially with the designed phase offsets, the robot loses propulsion and oscillates in place. This results in a structural limitation characterized by a persistent “stuck state,” preventing forward advancement [22].

This experiment was conducted to evaluate whether the proposed control algorithm can dynamically detect this stuck state and efficiently facilitate escape to ensure gait continuity. To control experimental variables, the stuck state was artificially induced by aligning all robot joints to the exact same phase angle prior to the initiation of the experiment. A comparative analysis was performed under identical initial conditions using the “Zero Model,” the “Rule-based Model,” and the “Main Model.” The primary performance metric was defined as the “escape time” required to fully exit the stuck state and return to a normal walking trajectory.

As indicated in Table 6, the Zero Model, lacking specific detection and correction algorithms, demonstrated a limitation in its inability to autonomously overcome the artificially induced stuck state across all ten experimental trials. The escape time for the Zero Model was recorded as “N/A” (Not Applicable) in every trial. This suggests that resolving a mechanical stuck state solely through simple repetitive motor actuation is highly challenging without controller intervention.

Unlike the Zero Model, the comparative Rule-based Model demonstrated attempts to recognize and escape the stuck state; however, its performance was limited. It failed to escape in Trials 3 and 7 (consistent with the failures observed in the walking experiment shown in Table 5). Even in the trials where it succeeded, it required an average of approximately 14.78 s, indicating a limitation in its ability to respond rapidly.

**Figure 9:**
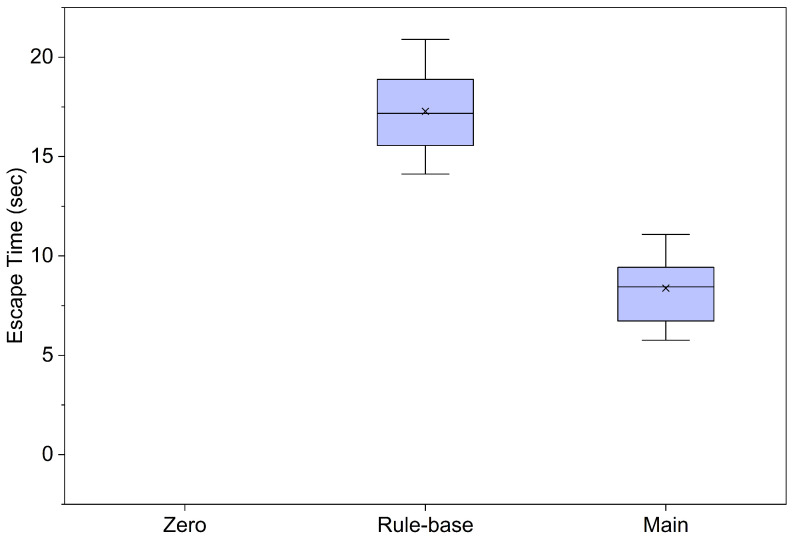
Box plot graph of Escape Time data. Symbol x represents the Mean. Detailed numerical mean and standard deviation (SD) statistics are provided in Table 6.

In contrast, the proposed Main Model successfully escaped the stuck state in all ten trials, exhibiting superior robustness. As illustrated in Figure 9, the Main Model recorded significantly lower escape times compared to the control groups—namely, the Zero Model (which failed to escape) and the Rule-based method. Furthermore, a paired *t*-test was conducted to verify the statistical significance of this improvement, yielding a result of p=0.0002<0.001, which confirms that the performance enhancement of the Main Model is statistically significant.

Specifically, the Main Model achieved a mean escape time of 8.41 s, with a minimum of 5.76 s recorded in Trial 4, demonstrating a recovery speed approximately 1.7 times faster than that of the Rule-based Model. These results clearly demonstrate that the proposed control architecture possesses an “autonomous recovery capability,” enabling it to detect constraint conditions in real-time based on current sensor information and actively resolve them to autonomously restore locomotive function.

### 4.3. Verification of Continuous Gait Stability and Linearity

This experiment was conducted to quantitatively analyze the impact of the proposed A-LSTM-based control algorithm on the robot’s “directional stability” and “linearity” during locomotion. Generally, robots based on the Klann linkage mechanism tend to drift in a specific direction or deviate from their intended trajectory under open-loop control (without feedback) due to mechanical tolerances or uneven ground friction. To verify the proposed method, we primarily evaluated locomotion efficiency by measuring the time required to traverse a 1 m distance, starting the robot from an identical initial posture. Furthermore, the robot’s ability to maintain a straight path was compared by analyzing the final displacement and path deviation observed during a 2 min continuous walking test.

According to Table 7, the Zero Model, from which feedback control was excluded, managed to travel an average of only 0.51 m over 2 min. As shown in Table 5, it exhibited unstable gait patterns, frequently failing to reach the 1 m target destination. This performance is attributed to the accumulation of imbalances in lateral propulsion during locomotion, causing the robot to deviate from the straight path, as well as its inability to autonomously compensate for velocity reductions caused by ground friction.

The comparative Rule-based Model recorded an average of approximately 0.67 m, demonstrating an improved travel distance of about 31% relative to the Zero Model. However, as observed in specific instances such as Trial No. 6 (0.1250 m), where the travel distance declined sharply under disturbance conditions, the model revealed a lack of locomotion consistency, indicating that fixed rules are insufficient to flexibly adapt to diverse environmental variables.

**Figure 10:**
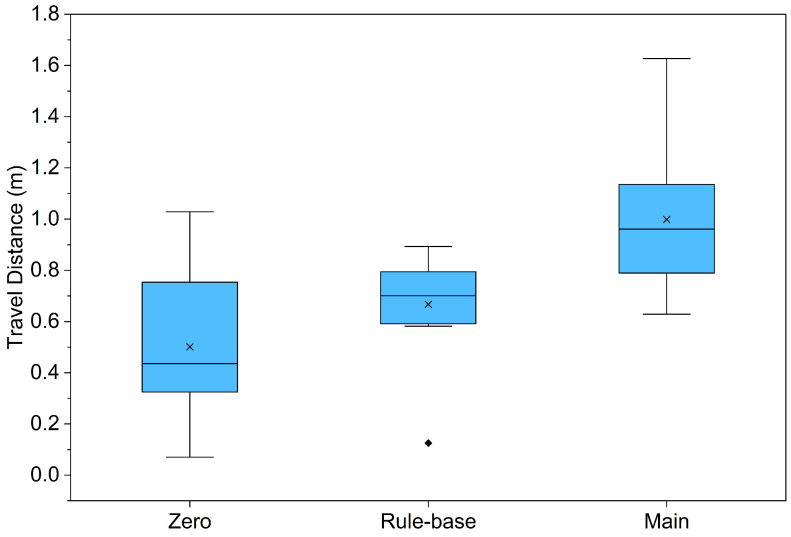
Box plot graph of Travel Distance data. Symbol x represents the Mean. Detailed numerical mean and standard deviation (SD) statistics are provided in Table 7.

In contrast, the Main Model, equipped with the proposed controller, traveled an average of approximately 1.00 m during the same duration. This represents a travel distance roughly double that of the Zero Model and about 1.5 times that of the Rule-based Model. Furthermore, a paired *t*-test was conducted to verify the statistical significance of these results, revealing that the performance improvement of the Main Model compared to the Rule-based Model is statistically significant (p=0.0041<0.01). Notably, in the 10th trial, a maximum distance of 1.63 m was achieved, demonstrating a substantial improvement in locomotion efficiency and stability. This performance is further supported by the statistical distribution shown in Figure 10, where the proposed model exhibits the highest mean and median values compared to the control groups. These results are attributed to the capability of the A-LSTM prediction-based PI controller to secure directional stability by correcting, in real-time, path deviations caused by asymmetric propulsion. Consequently, the findings of this study clearly demonstrate that practical and stable autonomous locomotion in real-world environments is achievable using solely the combination of low-cost current sensors and an AI model, without reliance on expensive position sensors.

The performance of the A-LSTM model is attributed to its ability to capture the kinematic phase-dependent current signatures of the Klann linkage. Specifically, the attention mechanism prioritizes current fluctuations at critical transition points in the gait cycle where mechanical resistance is non-linearly amplified. This suggests that the model has learned to approximate the complex, non-linear relationship between motor torque and linkage geometry, which is inherently difficult to define through deterministic rule-based logic, and this can be confirmed by experiments in Section 4.1, Section 4.2 and Section 4.3.

## 5. Conclusions and Future Work

To address the high cost and complexity associated with multi-degree-of-freedom walking robots, this study proposes and validates an AI-based sensorless feedback control framework that engineers the biological principle of proprioception. By leveraging the morphological intelligence of the Klann linkage, which mimics the walking mechanism of arthropods, an efficient hardware system was designed. Changes in motor current during locomotion were treated as muscle feedback signals and trained using an Attention-LSTM (A-LSTM) model. This approach enables real-time prediction of future motor states without additional external position sensors, confirming the potential of “interoceptive sensing”—the ability to infer a robot’s physical state solely through its internal electrical signal flow.

Experimental results demonstrate that the proposed system achieves precise gait control using only minimal internal sensory information. In terms of walking efficiency, the Main model successfully reached the destination in every trial of a 1 m straight walk, showing approximately a 35% improvement in mobility compared to the open-loop (Zero) model; this suggests that the performance stems from dynamic compensation via real-time state prediction rather than mere repetitive output execution. Notably, in stuck-state experiments designed to verify biological self-recovery capabilities, the proposed model proved its superior environmental adaptability by detecting abnormal states and normalizing gait within an average of 8.41 s. This signifies the successful engineering of a biological “reflex loop” mechanism, which detects anomalies and readjusts the gait phase solely through internal load variations. Furthermore, in long-term stability evaluations, the model actively compensated for surface disturbances and mechanical errors, achieving a travel distance approximately double that of the Zero model, indicating that AI-based phase inference effectively counteracts nonlinear variables such as terrain disturbances.

Although this study serves as an exploratory pilot, the statistically validated outcomes—demonstrated by a *p*-value of 0.0002 in recovery scenarios—establish a scalable paradigm for deploying high-level AI on resource-constrained biomimetic platforms. By bridging the gap between sophisticated deep learning and minimal sensing hardware, this work provides a foundation for future autonomous systems in unpredictable environments. In conclusion, this research academically and experimentally demonstrates that sophisticated gait perception and control are possible solely through the combination of current signals and AI models, without expensive external sensors.

In future research, we plan to advance the current angle prediction model by incorporating additional training data from conditions simulating real-world natural environments, such as slopes and irregular terrain. Furthermore, to align with the objective of low-cost commercialization, we aim to realize a “fully integrated, on-device biomimetic robot” by optimizing the A-LSTM model for Microcontroller Unit (MCU) environments. This will enable all sensory inference and control to occur locally on the robot without the need for external computing units, achieving a truly autonomous on-device architecture.

## Figures and Tables

**Table 1:** Link lengths and angles of the proposed mechanism.

Category	Link Index/Angle	Length/Angle Value
	$l_{1}$	1.81128
	$l_{2}$	3.42732
	$l_{3}$	6.67461
Link Length	$l_{3}^{\prime }$	4.55651
	$l_{4}$	4.71537
	$l_{5}$	9.49411
	$l_{5}^{\prime }$	9.52634
Angle	$l_{3}$ and $l_{3}^{\prime }$	169.994°
	$l_{5}$ and $l_{5}^{\prime }$	152.762°

**Table 2:** PI Control Parameters.

Parameter	Value	Description
Sampling Time ($T_{s}$)	0.1 s	Control loop period (10 Hz)
Proportional Gain ($K_{p}$)	0.4	Response gain for phase error
Integral Gain ($K_{i}$)	0.03	Gain for eliminating steady-state error
Filter Coefficient (α)	0.3	Error smoothing factor
Max Step Limit	±1.0%	Maximum duty cycle change per period

**Table 3:** Detail hyperparameter of AlSTM model.

Hyperparameter	Specification	Description
Model Architecture	Attention-LSTM	4 Layers, 128 Hidden Units
Batch Size	128	Mini-batch size
Optimization	Linear Decay	LR: 5 × 10^−5^ → 1 × 10^−5^
Loss Function	Combined MSE	MSE + Circular MSE (α=0.5)
Normalization	Standardization	Mean: 110, Std: 20
Epochs	5000	Total training iterations

**Table 4:** Summary of Modeling Parameters, Boundary Conditions, and Assumptions.

Category	Parameter	Value	Reference/Assumption
Mechanical	Link Length Ratios	see Table 1	Optimized via GA
	Fixed Link Angles	see Table 1	
	Total Mass	1.35 kg	Considered as a rigid body
Electrical	Rated Voltage	5 V	Constant supply assumed
	Sensor Resolution	0.1 mA	Linear calibration applied
	Sampling Frequency	10 Hz	Consistent with $T_{s}=0.1$ s
AI (A-LSTM)	Hidden Units/Layers	128/4	Optimized for time-series
	Input Window Size	30 steps	Captures periodic load patterns
	Loss Function	Circular MSE	Accounts for $0^{{}^{\circ}}/360^{{}^{\circ}}$ continuity
Control	$K_{p}$/$K_{i}$ Gains	0.4/0.03	Tuned for phase synchronization
	Slew Rate Limit	±1.0%	Prevents hardware damage

**Table 5:** Gait efficiency evaluation: omparison of 1 m travel time under open-loop and A-LSTM control.

Test Trial	Zero 1 m Travel Time (s)	Rule-Base 1 m Travel Time (s)	Main 1 m Travel Time (s)
No. 1	139.34 (2:19.34)	128.45 (2:08.45)	118.08 (1:58.08)
No. 2	Failed to Reach	145.12 (2:25.12)	137.22 (2:17.22)
No. 3	Failed to Reach	Failed to Reach	148.12 (2:28.12)
No. 4	151.88 (2:31.88)	156.33 (2:36.33)	161.96 (2:41.96)
No. 5	Failed to Reach	130.21 (2:10.21)	122.09 (2:02.09)
No. 6	Failed to Reach	135.67 (2:15.67)	126.92 (2:06.92)
No. 7	Failed to Reach	Failed to Reach	132.99 (2:12.99)
No. 8	Failed to Reach	105.90 (1:45.90)	90.28 (1:30.28)
No. 9	101.87 (1:41.87)	94.55 (1:34.55)	83.32 (1:23.32)
No. 10	Failed to Reach	72.40 (1:12.40)	60.09 (1:00.09)
Mean ± SD (s)	131.03±26.02	121.08±27.97	118.11±31.37

**Table 6:** Evaluation of autonomous recovery performance: Escape time across ten trials.

Test Trial	Zero Escape Time from Stuck State (s)	Rule-Base Escape Time from Stuck State (s)	Main Escape Time from Stuck State (s)
No. 1	N/A	15.34	9.27
No. 2	N/A	18.21	11.08
No. 3	N/A	N/A	6.73
No. 4	N/A	20.89	5.76
No. 5	N/A	16.45	9.43
No. 6	N/A	14.12	8.33
No. 7	N/A	N/A	8.25
No. 8	N/A	17.90	10.32
No. 9	N/A	19.56	6.09
No. 10	N/A	15.78	8.56
Mean ± SD (s)	N/A	17.28±2.27	8.38±1.76

**Table 7:** Evaluation of long-term walking stability: 2 min travel distance results.

Test Trial	Zero 2 min Travel Distance (m)	Rule-Base 2 min Travel Distance (m)	Main 2 min Travel Distance (m)
No. 1	0.9032	0.5821	1.0357
No. 2	0.35	0.7945	0.7897
No. 3	0.553	0.5912	0.7565
No. 4	0.7537	0.7023	0.6289
No. 5	0.3248	0.6540	0.9995
No. 6	0.4573	0.1250	0.9235
No. 7	0.07	0.6987	0.8232
No. 8	0.4136	0.8456	1.2643
No. 9	1.0283	0.7820	1.1357
No. 10	0.1592	0.8930	1.6274
Mean ± SD (m)	0.50±0.31	0.67±0.22	1.00±0.29

## Data Availability

The original contributions presented in this study are included in the article. Further inquiries can be directed to the corresponding author.

## Abbreviations

The following abbreviations are used in this manuscript:

AI	Artificial Intelligence
PWM	Pulse Width Modulation
CAD	Computer-Aided Design
DOF	Degree-Of-Freedom
IMU	Inertial Measurement Units
RNN	Recurrent Neural Networks
A-LSTM	Attention-based Long Short-Term Memory
MSE	Mean Squared Error
PI	Proportional-Integral
LPF	Low-Pass Filter
MCU	Microcontroller Unit
